# Satisfaction, Enjoyment and Boredom with Physical Education as Mediator between Autonomy Support and Academic Performance in Physical Education

**DOI:** 10.3390/ijerph17238898

**Published:** 2020-11-30

**Authors:** Raúl Baños, Julio Fuentesal, Luis Conte, María del Mar Ortiz-Camacho, Jorge Zamarripa

**Affiliations:** 1 Faculty of Physical Activity and Sports Sciences—INEF, Polytechnic University of Madrid, 28040 Madrid, Spain; raulfb89@gmail.com; 2 Faculty of Human and Social Sciences, Universidad Pontificia Comillas, 28049 Madrid, Spain; jfuentesal@comillas.edu; 3 Faculty of Sports Sciences, University of Murcia, 30720 Murcia, Spain; conte@um.es; 4 Faculty of Sports Sciences, Faculty of Educational Sciences, University of Granada, 18071 Granada, Spain; mmortiz@ugr.es; 5 Faculty of Sports Organization, Autonomous University of Nuevo Leon, 66451 San Nicolas de los Garza, Nuevo Leon, Mexico

**Keywords:** satisfaction, enjoyment, boredom, performance, autonomy support, physical education, secondary education, Mexico

## Abstract

The purpose of this study was to analyze the mediating effect of satisfaction/enjoyment and boredom between the perception of autonomy support and academic performance in physical education. The sample consisted of 374 girls (*M*_age_ = 13.99; *SD* = 0.30) and 374 boys (*M*_age_ = 14.02; *SD* = 0.33) from the state of Nuevo León, Mexico. The instruments used were the Questionnaire for Autonomy Support in Physical Education (CAA-EF), Sport Satisfaction Intrinsic in Physical Education (SSI-EF) and the physical education performance of the students. The instrument’s validity tests were analyzed using confirmatory procedures. Descriptive, reliability, and validity analyses were carried out for each instrument, and the mediating effect was examined; a mediation analysis was performed using the PROCESS V.3.5 macro. The main results revealed that autonomy support is not a direct indicator of physical education performance, but rather that students must feel satisfied with physical education for there to exist a forecast for a positive physical education performance. Satisfaction with physical education was found to have a mediating effect between autonomy support and physical education performance. However, boredom did not have a mediating effect between autonomy support and the student’s performance in physical education class.

## 1. Introduction

The poor academic performance of Mexican adolescents remains latent in the results from the Programme for International Student Assessment (PISA) 2018 Report. The academic level of secondary school students in Mexico is far inferior to the average for the countries of the Organization for Economic Cooperation and Development (OECD) (2018), having shown no improvement in this assessment since 2003. It is also important to mention that Mexicans scored very low as well in intrinsic satisfaction compared to the OECD average, a factor likely to affect the academic performance of adolescents [1].

Intrinsic satisfaction/enjoy may be examined according to the Theory of Subjective Well-Being [2]. This theory is composed of two dimensions: on the one hand, a person’s general satisfaction with life; and on the other hand, the affective dimension, understood as the result of immediate and continuous response to the conditions surrounding the students. In this way, satisfaction with school is interpreted as a cognitive-affective assessment of the general satisfaction experienced by the student [3]. It has been proven recently that levels of satisfaction with physical education (PE) forecast satisfaction with school, which is linked in turn to the academic performance of secondary school students [4,5]. But what strategies can the PE teacher apply to increase the levels of satisfaction with PE?

One way for the teacher to increase levels of satisfaction with PE is by supporting the autonomy of their students [6]. Autonomy support—together with competence and relationship—is one of the dimensions that form the Theory of Basic Psychological Needs [7], variables that have been studied extensively [8,9]. Autonomy refers to the level of independence and control that an individual has over their own choices; competence is defined as a person’s capability to perform a certain task; and relationship means the connection that an individual has with other persons [10].

Autonomy, understood as a psychological disposition before a certain task, is vital in the educational environment, and especially in the field of PE, as it is linked to individual competences that are reinforced through teamwork. Instructions or orders for practical tasks from the teacher to the student encourage their autonomy and improve their concentration [11,12]. Moreover, as explained in Ntoumanis’ research [13], the fulfillment of autonomy increases academic commitment, favors motivation, and prevents task abandonment and dropout rates in general [11]. According to Huescar, Fabra, and Moreno-Murcia [14], a broader autonomy support coming from the teacher, family, and peers is connected to the fulfillment of basic psychological needs, improved self-determined motivation, greater perceived control, a positive mindset and the intention to engage in physical activity. 

Autonomy brings a perception of competence, capacity, and control over an individual’s own decisions, which implies accepting the consequences of their behavior. Such perceptions of freedom and competence enable the student to face more complex challenges, generating more in-depth learning [15].

In this sense, fulfilling one’s need for autonomy with regard to decisions and behaviors translates into an improved psychological well-being. In the educational field, nonetheless, autonomy is equally relevant for the learning process, given that the student’s decisions come together with exercising various capabilities that teachers shall encourage [16]. The teacher’s behavior in the classroom in terms of autonomy support is paramount in order to help achieve higher levels of motivation [17].

In this regard, an individual fulfilling their basic psychological needs (autonomy, competence, and relationship) will see their satisfaction with and commitment to ongoing activities increase [7], for example, their levels of physical activity during leisure time [18], which in turn translate into an improvement in their academic performance [19,20]. It is worth underscoring how important it is that students feel satisfied with PE class so that their levels of physical activity during their leisure time increase [21].

According to this, it is critical that students enjoy and feel satisfied with the PE class due to its connection to autonomy support [6], academic performance [5], and physical activity levels [21], which in turn relate to better academic performance [22]. On the contrary, when a student feels unsatisfied/boredom with PE class, their levels of physical activity decline [21], and their levels of school boredom [23] and potential school abandonment increase [24], among other variables.

For all that, the purpose of this study is to analyze the mediating effect of satisfaction/enjoyment and boredom between the perception of autonomy support and academic performance in PE.

## 2. Materials and Methods

### 2.1. Design and Type of Research

This study features a non-experimental, cross-sectional and correlational-causal design [25]. This research was carried out in accordance with the 1961 Declaration of Helsinki (Edinburgh revision, 2000). Approval was obtained from the Secretaría de Educación Pública of Mexico (identification number: 431/569/E) and The Universidad Autónoma de Baja California, Mexico.

### 2.2. Participants

Sample design was probabilistic by centers, stratified, multistage, and by proportional affixation, comprised of third-grade secondary school students from Nuevo León, Mexico. Participant schools were selected at random. The number of third-grade secondary school students in the state of Nuevo León was 13,396 girls and 13,831 boys. A representative sample was calculated according to sex for a finite population with a confidence level of 95% and a margin of error of +5%, consisting of 374 girls (*M*_age_ = 13.99; *SD* = 0.30) and 374 boys (*M*_age_ = 14.02; *SD* = 0.33).

### 2.3. Instruments

The questionnaire used was comprised of the following scales. To measure autonomy support from the PE teachers, we used the CAA-EF validated for the Mexican context by Maldonado, Pacheco and Zamarripa [26]. This questionnaire was adapted from the Learning Climate Questionnaire by Williams and Deci (1996), based in turn on the Health-Care Climate Questionnaire [27], which contains 15 items to measure the professor’s support for autonomy through one dimension: autonomy support. The guideline is to grade according to the items; answers are given on a seven-point scale of polytomous items ranging from 1 (strongly disagree) to 7 (strongly agree). In previous studies with a Mexican sample the CAA-EF had acceptable fit indexes (NNFI = 0.99, CFI = 0.99 and RMSEA = 0.06) and the internal consistency (α = 0.92) was acceptable [26]. 

To measure intrinsic satisfaction (enjoyment and boredom) in PE, we used the Sport Satisfaction Intrinsic in Physical Education (SSI-EF) instrument adapted to the Mexican context by Baños et al. [4]. The instrument is composed of eight items; five of them measure the level of satisfaction/enjoyment with academic activities for each subject, while the remaining three measure boredom. The scale is preceded by the phrase: “Tell us how much you agree or disagree with physical education”. Answers were given using a Likert scale ranging from 1 (completely disagree) to 5 (strongly agree). In previous studies with a Mexican sample the SSI-EF had acceptable fit indexes (NNFI = 0.96, CFI = 0.97 and RMSEA = 0.04) and the alpha values found were 0.78 for the satisfaction/enjoyment subscale and 0.65 for the boredom subscale [4]. 

Academic performance in PE. We asked teachers to provide their latest test score records in order to analyze the students’ grades. This procedure guarantees a better reliability than the one used by Baños et al. [5], where grades were requested from the students themselves. These were recorded in a scale of polytomous items ranging from 1 to 10.

### 2.4. Procedure

In order to carry out this study, a research project called “Programme for International Student Assessment: relationship between school performance in secondary school students and psychological, family, and physical activity variables” was first presented to, and later approved and subsidized by the Secretaría de Educación Pública. Then, authorization was requested from school principals, providing the parents/guardians involved with information for consent detailing the purpose and intentionality of the study. Following their approval, the data collection procedure began by informing the participants of the study’s purpose, that participation was anonymous and voluntary, and that their answers were to remain confidential, reminding them that there were no right or wrong answers, and asking them to answer with complete honesty. All questionnaires were filled inside the classroom in the presence of the lead researcher in case of doubts during the procedure, which lasted 15–20 minutes. The data was collected in September 2019.

### 2.5. Statistical Analysis

Initially, an analysis for multivariate normality was performed. To that end, we conducted a normality test based on the PRELIS relative multivariate kurtosis test (RMK) using the LISREL 8.80 software. Once normality was determined, or not, a confirmatory factorial analysis (CFA) was carried out to assess the proper adequacy of these instruments in regard to the samples used in this research. Several reliability and validity indices were calculated for each instrument, including Cronbach’s alpha, composite reliability, and average variance extracted (AVE). Then, analyses were carried out to determine the correlation among the instruments used. Subsequently, several structural equation models were created in order to meet the purpose of this study. Statistical Package for the Social Sciences SPSS v.22 (IBM, Armonk, NY, USA) and the Linear Structural Relations (LISREL) V.8.80 software (Scientific Software International, Inc., Lincolnwood, IL, USA) were used for these calculations.

In addition, a mediation analysis (model 4) was carried out in the PROCESS V.3.5 macro (www.processmacro.org) [28] for the SPSS Statistics software V.21 (IBM, Armonk, NY, USA) to determine whether the correlation between autonomy support and PE grades was mediated by enjoyment and boredom during class. Confidence intervals (95%) were generated using a bootstrap of 10,000 samples to determine the outcomes of the model. Likewise, we calculated the indirect effects of autonomy support (X) and PE grades (Y) through enjoyment (M1) and boredom (M2).

## 3. Results

### 3.1. Data Normality Analysis

Table 1 shows the normality data of the measurement instruments, where data finally show a non-normal behavior. The RMK values were 1.380 for CAAEF and 1.805 for SSI-EF.

### 3.2. Confirmatory Factor Analysis

First, CFA were performed for each instrument to confirm their validity and reliability in regard to the sample expected to be used in this research. Results proved (Table 2) to be acceptable within the threshold established in χ^2^/df [29,30], in the goodness of fit index (GFI) [31], comparative fit index (CFI), normed fit index (NFI), non- normed fit index (NNFI) [32], and in the root mean square error of approximation (RMSEA) [33,34].

### 3.3. Reliability and Validity Analysis

Table 3 shows an analysis for each model with the values for Cronbach’s alpha, composite reliability, and average variance extracted (AVE). As can be seen, all reliability, AVE and almost all α indices exceed the acceptable threshold according to Dunn, Baguley, and Brunsden [35] and Hair, Black, Babin, and Anderson [36]. However, because of the limited number of items per factor (as is the case with the boredom dimensions), α ≤ 0.70 values may be deemed acceptable [37]. Moreover, it must be kept in mind that composite reliability is preferred over Cronbach’s alpha in ordinal data scales, as the former does not depend on the number of attributes associated to each concept [38].

### 3.4. Correlation Analysis

Table 4 shows how perceived autonomy support correlates positively and significantly with satisfaction/enjoyment (0.526 **) and PE score (0.122 **), and negatively with boredom in PE (−0.087 *). satisfaction/enjoyment with PE correlated positively with PE score (0.170 **) and negatively with boredom (−0.259 **). Boredom in PE was negatively correlated with PE score (−0.085 *).

### 3.5. Mediating Effect

The proposed model calculated the effect of mediation from satisfaction/enjoyment (M1) and boredom (M2) on the interaction between autonomy support (X) and the grades obtained in PE class (Y).

**Figure 1:**
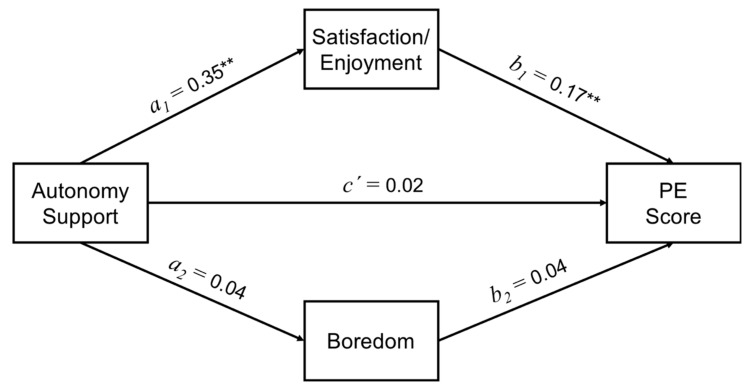
Statistical diagram of the satisfaction/enjoyment and boredom as mediators between autonomy support with PE score. ** *p* < 0.001.

Results revealed that autonomy support was positively and significantly correlated with satisfaction/enjoyment in PE class (*a*_1_ = 0.351; *p* < 0.001), however, this was not the case with boredom (*a*_2_ = 0.042; *p* = 0.113). Likewise, enjoyment and PE grades were positively and significantly correlated (*b*_1_ = 0.166; *p* < 0.001), contrary to the correlation between boredom and PE grades, which was not significant (*b*_2_ = 0.041; *p* = 0.212) (see Table 5 and Figure 1).

As shown in Table 5, the interaction between autonomy support and PE grades was not mediated by boredom (IE = 0.002; IC = −0.0014; 0.0064). However, enjoyment had an indirect, positive and significant effect (IE = 0.058; IC = 0.0270; 0.0917) on the interaction between autonomy support and PE grades. 

## 4. Discussion

The purpose of this study was to analyze the mediating effect of satisfaction/enjoyment and boredom between the perception of autonomy support and academic performance in PE. The psychometric properties of CAA-EF and SSI-EF supported the reliability and validity of both scales. Similar results were achieved by other researchers, both in the Mexican context for CAE-EF [26] and SSI-EF [24], as well as in their original version for CAA-EF [39] and SSI-EF [40].

In terms of the correlation analysis, the results obtained confirm a positive correlation among autonomy support, satisfaction/enjoyment, and PE score, although the correlation scores obtained are low. On the contrary, boredom during PE class correlates negatively with autonomy support and PE score. In this sense, other studies found a correlation between autonomy support from the PE teacher and intrinsic motivation, and the sense of competence and autonomy from the students [17], positive correlations between students and satisfaction with PE class [8,41,42]. Moreover, it has been demonstrated that a student’s satisfaction with PE class forecasts a positive PE score [4,5]. Therefore, autonomy support may increase satisfaction with PE and keep the student from boredom, also increasing motor engagement in the classroom and adherence to extracurricular sport activities [6].

Said positive correlation between autonomy support, satisfaction/enjoyment, and PE performance might be a result of responsibility being transferred from the teacher to the students, thus allowing them to make their own decisions while acknowledging personal effort and self-improvement, as well as the spectrum of activities to perform during PE class [8,41,43,44]. Other studies have revealed that when teachers choose to exercise autonomy support over strategies for control, the students are more likely to participate in proposed tasks, exhibit a greater commitment to their activities and perceived competence, and feel more satisfied with their lives [45].

The main focus of this study was to analyze the mediating role of satisfaction/enjoyment and boredom with PE in the correlation between the perception that students have about the PE teacher’s support for autonomy and the academic performance achieved in the subject. In terms of the mediating effects over satisfaction/enjoyment and boredom with PE, satisfaction/enjoyment with the PE subject had a mediating effect between the academic performance achieved in the class and the teachers’ support for autonomy. However, boredom with PE had no significant results on the mediating effect between autonomy support and academic performance. In this regard, Trigueros et al. [46] found that satisfaction with PE had a mediating effect between autonomy support in PE and the fulfillment/unfulfillment of basic psychological needs during physical activities. The results of this study make an important contribution to scientific literature. In order to see the student’s academic performance increase in PE, the teacher should adopt not only an autonomy-supportive style, but also strategies to make the students enjoy and feel satisfied with PE class [47]. However, several studies have shown that levels of satisfaction/fun in PE class are lower in Mexican adolescents compared to students from other countries [48]. For this reason, it is important that teachers create fun and novel sessions, moving away from monotonous and boring classes [23,49,50].

It is worth mentioning that several researchers have demonstrated that students who engage in moderate and vigorous physical activity achieve better academic performance in mathematics and reading comprehension [19,22,51]. Nonetheless, although other studies have not found any significant correlations among these variables, physical activity levels did not negatively affect academic performance [52,53]. An explanation for this discrepancy in the scientific literature might lie on the scarcity of studies analyzing the mediating effect of autonomy support and satisfaction with PE between levels of physical activity and academic performance. Therefore, it would be interesting to see future researchers elaborate further on this field.

## 5. Limitations

This study faced some limitations which must be kept in mind, among which is the fact that only one dimension from the theory of basic psychological needs was considered (autonomy). For future research, it is recommended to analyze the other dimensions pegged to this theory as well (sense of competence and relationship with peers). However, in spite of these limitations, some strengths of this research shall be highlighted. The sample selection was probabilistic and random by centers, stratified, multistage, and by proportional affixation. Therefore, the study can be generalized for the state of Nuevo León, Mexico. In addition, this research topic may help create solutions for the key issues faced by teachers every day.

## 6. Conclusions

As a conclusion, the results of this study have shown that autonomy support does not directly predict PE performance, but rather that it is necessary that students feel satisfied with PE for there to be a positive forecast of the student’s academic performance. In this way, satisfaction with PE has a mediating effect between autonomy support and academic performance. However, boredom with PE did not have a mediating effect between autonomy support and the student’s academic performance.

## Figures and Tables

**Table 1:** Values of multivariate normality test.

Instrument	Multivariant Normalized Kurtosis	Mardia-Based Kappa	Higher Limit	Lower Limit
CAA-EF	58.8716	0.38	1.018	0.983
SSI-EF	69.8543	0.805	1.032	0.968

**Table 2:** Adjustment indices of each model.

Instrument	χ^2^	df	χ^2^/df	*p*	GFI	CFI	NFI	NNFI	RMSEA
CAA-EF	303.66	90	3.37	0.000	0.99	0.98	0.97	0.98	0.056
SSI-EF	34.30	19	1.80	0.016	0.99	0.97	0.94	0.96	0.040

Note. GFI = goodness of fit index, CFI = comparative fit index, NFI = normed fit index, NNFI = non-normed fit index, RMSEA = root mean square error of approximation.

**Table 3:** Scale of reliability and composite validity.

Variable	*M*	*SD*	95% CI		IQR	CR	AVE	α
Autonomy Support	4.07	1.53	3.96	4.18	2.27	0.93	0.50	0.95
Satisfaction/Enjoyment	3.68	1.01	3.60	3.74	1.50	0.89	0.63	0.84
Boredom	2.48	1.13	2.40	2.57	1.60	0.67	0.55	0.68

Note. M = mean; SD =standard deviation; 95% CI = confidence interval; IQR = interquartile range; CR = composite reliability; AVE = average variance extracted.

**Table 4:** Correlation analysis.

Variable	1	2	3
1. Autonomy Support			
2. Satisfaction/Enjoyment	0.526 **		
3. Boredom	−0.087 *	−0.259 **	
4. PE score	0.122 **	0.170 **	−0.085 *

Note. ** *p* < 0.01, * *p* < 0.05.

**Table 5:** Regression coefficients, standard errors, and model summary information for the mediational effects of satisfaction/enjoyment and boredom in the relationship between autonomy support and PE score.

Antecedent	*M*_1_ (Satisfaction/Enjoyment)			*M*_2_ (Boredom)			*Y* (PE Score)			
	Coeff.	SE	*p*	Coeff.	SE	*p*	Coeff.	SE	*p*	IE
*X*_(Aut. Sup.)_	0.351	0.024	<0.001	0.042	0.026	0.113	0.022	0.027	0.432	
*M*_1 (Satisfaction/Enjoyment)_							0.166	0.043	<0.001	0.058 *
*M*_2(Boredom)_							0.041	0.033	0.212	0.002
Constant	2.246	0.086	<0.001	3.353	0.115	<0.001	8.223	0.165	<0.001	
	*R*^2^ = 0.284			*R*^2^ = 0.0003			*R*^2^ = 0.041			
	*F*_(1, 751)_ = 297.548, *p* < 0.001			*F*_(1, 751)_ = 2.523, *p* = 0.113			*F*_(3, 749)_ = 10.676, *p* < 0.001			

Note. Aut. Sup = autonomy support; PE = physical education; Coeff. = coefficient; IE = indirect effect. * = indirect effect significant (confidence intervals do not include zero).
